# Supersized Atheroma Causing Acquired Coarctation of Aorta Leading to Heart Failure

**DOI:** 10.1177/2324709616689477

**Published:** 2017-01-01

**Authors:** Sajin Karakattu, Ghulam Murtaza, Sharma Dinesh, Kamesh Sivagnanam, Jeffrey Schoondyke, Timir Paul

**Affiliations:** 1 East Tennessee State University, Johnson City, TN, USA

**Keywords:** coarctation of aorta, calcification, atheromatous lesion

## Abstract

Calcified atheromatous aortic lesion causing significant narrowing of the aorta is an uncommon clinical entity. This calcified atheroma leads to obstruction of the lumen of the aorta simulating acquired coarctation of aorta causing impaired perfusion of lower limbs, visceral ischemia, and hypertension. We report a case of 58-year-old patient who presented with dyspnea on exertion, orthopnea, paroxysmal nocturnal dyspnea, 25-lb weight gain, lower extremity edema, and chest pain. Extensive workup including computed tomography and magnetic resonance imaging revealed a large calcific mass in the aortic arch causing his presenting symptoms. After surgical correction his symptoms resolved. Any patient presenting with heart failure symptoms in the setting of uncontrolled renovascular hypertension, intermittent claudication symptoms, or visceral ischemia with normal ejection fraction but moderate to severe left ventricular hypertrophy should be in high suspicion for acquired coarctation of aorta. The routine thorough examination of pulses in bilateral upper and lower extremities in all hypertensive patients is a very simple and useful clinical tool to diagnose acquired aortic coarctation.

## Introduction

Acquired atheromatous coarctation of aortic arch is an uncommon finding with an estimated prevalence between 0.6% and 1.8%. Occlusive atherosclerotic disease usually involves the infrarenal aorta and the aortic bifurcation. Localized obstruction in a suprarenal aorta of normal diameter caused by an eccentric, heavily calcified lesion is uncommon. This heavily calcified plaque expands into the lumen and can cause significant narrowing, which may lead to malperfusion of the lower limbs, cause visceral ischemia distal to obstruction, heart failure due to increased afterload, and hypertension due to renal ischemia and/or aortic luminal obstruction.^1^

## Case Report

**Figure 1.**
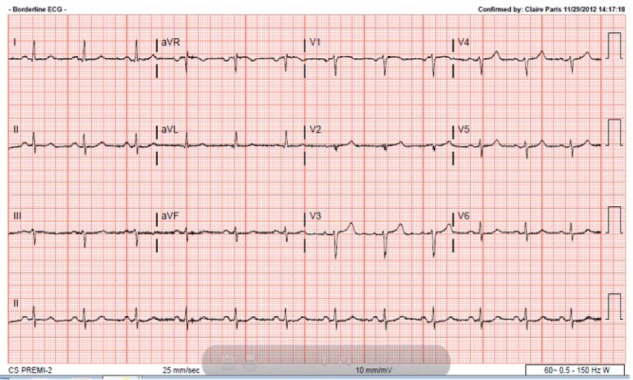
Poor R wave progression and low-voltage QRS in pre-cordial leads.

**Figure 2.**
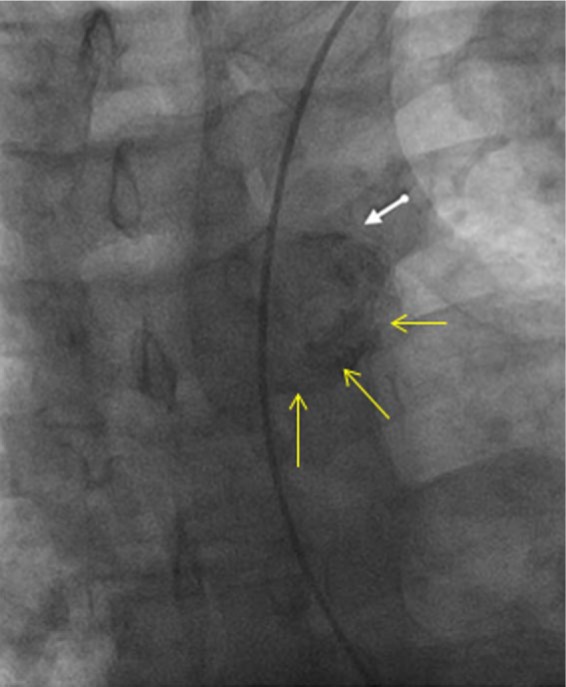
Arrows indicating radiolucency around aortic arch by fluoroscopy.

**Figure 3.**
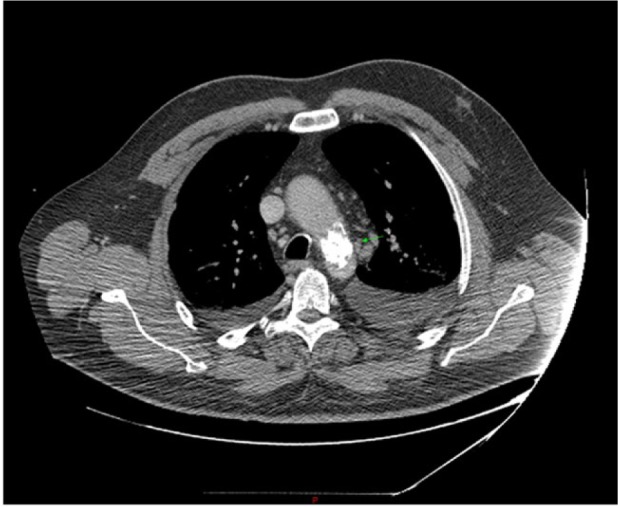
Calcified aortic lesion near aortic arch by computed tomography angiography.

**Figure 4.**
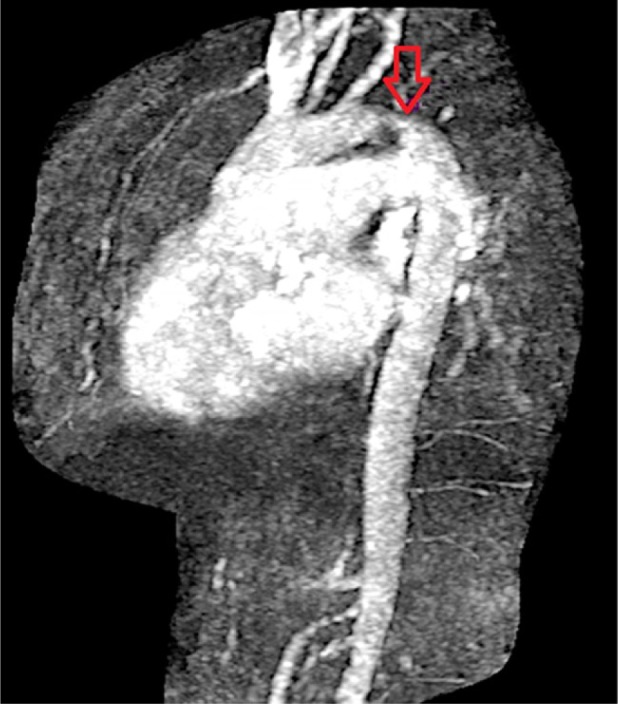
High-grade stenosis caused by calcified plaque just distal to subclavian artery indicated by arrow revealed in magnetic resonance angiography.

A 58-year-old male with medical history significant for hypertension, type 2 diabetes mellitus, morbid obesity, hyperlipidemia, and 35 pack-year of smoking history presented with chief complaint of dyspnea on exertion and paroxysmal nocturnal dyspnea for 2 weeks. He was being followed by his primary care physician for uncontrolled blood pressure. His home medications included aspirin 81 mg once daily, hydralazine 100 mg 3 times daily, amlodipine 10 mg once daily, carvedilol 25 mg twice daily, and minoxidil 20 mg twice daily. He reported that he has been having progressive difficulty in breathing, worsening lower extremity edema, orthopnea, dry cough, and 25-lb weight gain in 2 weeks. His blood pressure at presentation was 172/64 mm Hg, pulse rate 86 beats per minute, respiratory rate 18 breaths/min, and oxygen saturation 92% on 2 L of oxygen. He had 1+ lower extremity edema with very weak bilateral lower extremity pulses. Laboratory evaluation was remarkable for mildly elevated troponin at 0.1 ng/mL along with BNP of 231 pg/mL. Chest X-ray did not show any infiltrates. Heart size was normal and lung fields were clear. Electrocardiogram showed poor R wave progression and low voltage QRS in pre-cordial leads (Figure 1). Transthoracic echocardiogram showed severe left ventricular hypertrophy, left ventricular ejection fraction of 55% to 60%, and grade II diastolic dysfunction. He was started on IV Lasix and taken to the cardiac catheterization laboratory with a working diagnosis of acute coronary syndrome. There was difficulty in advancing the guidewire across the aortic arch. Thus, fluoroscopy was performed, which showed a large radiolucency around the aortic arch (Figure 2). Next, brachial access was obtained and left heart catheterization and coronary angiogram was performed that showed moderate nonobstructive coronary artery disease. Urgent computed tomography (CT) chest angiogram showed focal globular calcification near the distal arch of aorta (Figure 3) causing near complete occlusion leading to acquired aortic coarctation. The vascular anatomy was further delineated by magnetic resonance imaging (MRI) chest angiogram that revealed focal stenosis greater than 75% causing narrowing in the aortic arch just distal to the origin of subclavian artery (Figure 4). The abdominal aorta was normal in caliber with no evidence of dissection or aneurysm. The patient was also found to have low ankle brachial index performed due to diminished pulses in lower extremities. He underwent ascending aortic to proximal descending aorta bypass with a 16 mm Hemashield graft approached through median sternotomy with left posterior lateral thoracotomy. He tolerated the procedure well and had an uneventful discharge from the hospital. On 3 months follow-up with cardiology office, he reported improved exercise tolerance with markedly improved shortness of breath and claudication symptoms.

## Discussion

Coarctation of the aorta is a narrowing of aorta typically located at the insertion of the ductus arteriosus just distal to the left subclavian artery. It is generally a congenital anomaly accounting for 4% to 6% of all congenital heart defects.^2^ Acquired aortic narrowing is usually seen in inflammatory conditions of aorta such as Takayasu arteritis. But acquired coarctation due to extensive calcified plaque creating a mass-like appearance causing stenosis of aorta is uncommon with unclear etiology.

This disease entity was first described in 1984 by Qvarfordt et al with obstructive lesions of the suprarenal aorta, which was named as “coral reef aorta” because the lesions resembled those of oceanic structures^1,3^ based on its irregular, gritty, whitish hard-rock mass-like appearance in aorta. This calcification expands gradually in the luminal part of the aorta that resembles the growth of hyperplastic bone with no abnormalities in serum calcium.

In a series of 70 patients over a period of 23 years, prevalence of this disease entity was between 0.6% and 1.8%.^4^ Sex distribution was found to be nearly equal 55.3% in women and 44.7% in men with median age of 59.5 years. Majority of these patients have risk factors with strong predisposition to arteriosclerosis including heavy smoking, hypertension, dyslipidemia, and diabetes.

The pathogenesis of these atherosclerotic calcifications is not well understood yet. It has been suggested that this unique phenomenon may be due to calcification of fibrin platelet thrombus. This is supported by the fact that inhibitors of vascular calcifications such as fetuin-A and uncarboxylated matrix gla protein are decreased in these patients, suggesting the development of calcified mass.^5^ The common risk factors for atherosclerotic cardiovascular diseases, such as smoking, dyslipidemia, and arterial hypertension, are not sufficient to explain this pathogenesis. There have been some assumptions that disturbances of calcium metabolism and infectious or inflammatory processes occurring in the vessel wall could be responsible for these calcifications, but this has not been proven in large studies yet.^3,7^ In general, aortic atherosclerosis commonly involves the aortic bifurcation, while the coral reef aortic calcification occurs in suprarenal aorta^3^ beyond the aortic arch with rare involvement of arch.

The symptoms and clinical presentation of this large calcific atheromatous plaque depends on the extent of disease and the location of the aortic segment involved. These heavily calcified plaques can cause significant luminal stenosis, which may lead to malperfusion of the lower limbs, difference in blood pressure between upper and lower extremities, visceral ischemia, heart failure due to increased afterload, or hypertension due to renal ischemia. Usual clinical presentation is bilateral lower limb claudication, renovascular hypertension, abdominal angina with symptoms of nausea, vomiting, abdominal pain, weight loss, symptoms due microvascular embolization in involved organ distally, impaired renal function, and subsequently end-stage renal disease.^4,6-8^ To our knowledge there have been only 2 case reports of congestive heart failure as initial presentation of this entity. In these cases, calcifications were located mainly in descending thoracic and abdominal aorta.^9,10^ To the best of our knowledge, this is the first reported case of congestive heart failure due to calcified mass involved in aortic arch. The underlying mechanism of congestive heart failure is afterload mismatch due to stenotic proximal aorta and activation of renin-angiotensin-aldosterone system due to renal ischemia. Fortunately, in our case the patient was diagnosed and treated at an earlier stage so he did not develop any serious complications of malperfusion of kidneys, viscera, or lower extremities.

Due to wide variety of clinical presentation depending on the extent of disease and location of aorta, high index of clinical suspicion is required. The location of this atherosclerotic lesion is a valuable diagnostic information, because this calcified atherosclerotic mass tends to be seen primarily in the juxtarenal and suprarenal locations.^11^ Commonly CT and/or MR angiogram of the entire aorta in anteroposterior and lateral views are necessary for diagnosis, extent of calcification, and defining the optimal site of aortic clamping for surgical management. In a patient with a proven pressure gradient, total angiographic visualization of the aorta should be performed.

Treatment options are transaortic thromboendarterectomy or placement of a thoracoabdominal bypass graft in accordance with the localization of the stenosis.^12,13^ Early release of aortic obstruction is critical to prevent irreversible end-organ damage and resolution of symptoms. Thromboendarterectomy is the treatment of choice in majority of the cases. A bypass can be done if the lesion extends too far into the visceral and/or renal arteries. Stent implantation can be considered in patients with favorable anatomy.^14,15^ When the quality of the affected segment cannot be ensured after the procedure, an open graft replacement is required.^1^ Our patient reported improved exercise tolerance with markedly improved shortness of breath and claudication symptoms on follow-up after surgical correction of this aortic mass.

Early diagnosis and treatment of this calcified lesion is important; otherwise, life-threatening renal and visceral complications and irreversible organ damage can develop. Significant delay between the development of symptoms and surgical treatment might happen if physicians fail to recognize the kind and extent of disease.

## Conclusion

Any patient who presents with heart failure symptoms and hypertension with normal ejection fraction but moderate to severe left ventricular hypertrophy, acquired coarctation of aorta should be in differential diagnosis. Concomitant claudication symptoms, visceral ischemia, and difficult to control hypertension can serve as important clues. Early diagnosis and treatment is important; otherwise, life-threatening renal and visceral complications can develop. Due to the wide variety of clinical presentations depending on the extent of disease and location of aorta, high index of clinical suspicion is required. The routine thorough examination of pulses in bilateral upper and lower extremities, especially radio-femoral delay in all hypertensive patients, is a very simple and useful clinical tool to diagnose acquired aortic coarctation.
